# Supplementary material for: Congenital disorder of glycosylation caused by starting site-specific variant in syntaxin-5
Source: Nat Commun. 2021 Oct 28;12:6227. doi: 10.1038/s41467-021-26534-y (PMC8553859; doi:10.1038/s41467-021-26534-y)
Supplement: Supplementary file 3 — Description of Additional Supplementary Files. [file 41467_2021_26534_MOESM3_ESM.pdf]

Description of additional supplementary files

Title: Supplementary Data 1.

Description: Microarray data.

Title: Supplementary Movie 1.

Description: Epifluorescence time-lapse imaging of RUSH cargo ManII-SBP-EGFP (green) trafficking in wildtype HeLa after biotin addition. Magenta: Giantin-mScarlet.

Title: Supplementary Movie 2.

Description: Epifluorescence time-lapse imaging of RUSH cargo ManII-SBP-EGFP (green) trafficking in HeLa Stx5ΔL HeLa after biotin addition. Magenta: Giantin-mScarlet.

Title: Supplementary Movie 3.

Description: Epifluorescence time-lapse imaging of RUSH cargo VSVG-ts045-EGFP (green) trafficking in wildtype HeLa after biotin addition. Magenta: Giantin-mScarlet.

Title: Supplementary Movie 4.

Description: Epifluorescence time-lapse imaging of RUSH cargo VSVG-ts045-EGFP (green) trafficking in Stx5ΔL HeLa after biotin addition. Magenta: Giantin-mScarlet.

Title: Supplementary Movie 5.

Description: Epifluorescence time-lapse imaging of RUSH cargo Stx5S-SBP-mCitrine (green) trafficking in wildtype HeLa after biotin addition. Magenta: Giantin-mScarlet.

Title: Supplementary Movie 6.

Description: Epifluorescence time-lapse imaging of RUSH cargo Stx5L-SBP-mCitrine (green) trafficking in wildtype HeLa after biotin addition. Magenta: Giantin-mScarlet.

Title: Supplementary Movie 7.

Description: Epifluorescence time-lapse imaging of RUSH cargo Stx5LΔER-SBP-mCitrine (green) trafficking in wildtype HeLa after biotin addition. Magenta: Giantin-mScarlet.
