# Supplementary material for: Congenital disorder of glycosylation caused by starting site-specific variant in syntaxin-5
Source: Nat Commun. 2021 Oct 28;12:6227. doi: 10.1038/s41467-021-26534-y (PMC8553859; doi:10.1038/s41467-021-26534-y)
Supplement: Supplementary file 12 — Reporting summary. [file 41467_2021_26534_MOESM12_ESM.pdf]

nature research

Corresponding author(s): Geert van den Bogaart, Dirk Lefeber

Last updated by author(s): Sep 4, 2021

Reporting Summary

Nature Research wishes to improve the reproducibility of the work that we publish. This form provides structure for consistency and transparency in reporting. For further information on Nature Research policies, see our Editorial Policies and the Editorial Policy Checklist.

Statistics

For all statistical analyses, confirm that the following items are present in the figure legend, table legend, main text, or Methods section.

n/a	Confirmed	
☐	☒	The exact sample size (n) for each experimental group/condition, given as a discrete number and unit of measurement
☐	☒	A statement on whether measurements were taken from distinct samples or whether the same sample was measured repeatedly
☐	☒	The statistical test(s) used AND whether they are one- or two-sided<br>*Only common tests should be described solely by name; describe more complex techniques in the Methods section.*
☒	☐	A description of all covariates tested
☐	☒	A description of any assumptions or corrections, such as tests of normality and adjustment for multiple comparisons
☐	☒	A full description of the statistical parameters including central tendency (e.g. means) or other basic estimates (e.g. regression coefficient) AND variation (e.g. standard deviation) or associated estimates of uncertainty (e.g. confidence intervals)
☐	☒	For null hypothesis testing, the test statistic (e.g. F, t, r) with confidence intervals, effect sizes, degrees of freedom and P value noted<br>*Give P values as exact values whenever suitable.*
☒	☐	For Bayesian analysis, information on the choice of priors and Markov chain Monte Carlo settings
☒	☐	For hierarchical and complex designs, identification of the appropriate level for tests and full reporting of outcomes
☐	☒	Estimates of effect sizes (e.g. Cohen's d, Pearson's r), indicating how they were calculated

Our web collection on statistics for biologists contains articles on many of the points above.

Software and code

Policy information about availability of computer code

Data collection	Data was collected using the software from the microscopes (mainly Leica LAS X software) and analyzed by the open source software FIJI ImageJ
Data analysis	ImageJ macros for the trafficking experiments can be requested.

For manuscripts utilizing custom algorithms or software that are central to the research but not yet described in published literature, software must be made available to editors and reviewers. We strongly encourage code deposition in a community repository (e.g. GitHub). See the Nature Research guidelines for submitting code & software for further information.

Data

Policy information about availability of data

All manuscripts must include a data availability statement. This statement should provide the following information, where applicable:

- Accession codes, unique identifiers, or web links for publicly available datasets
- A list of figures that have associated raw data
- A description of any restrictions on data availability

All primary data is available on request. Constructs will be deposited at the open repository Addgene.

Field-specific reporting

Please select the one below that is the best fit for your research. If you are not sure, read the appropriate sections before making your selection.

☒ Life sciences ☐ Behavioural & social sciences ☐ Ecological, evolutionary & environmental sciences

For a reference copy of the document with all sections, see nature.com/documents/nr-reporting-summary-flat.pdf

Life sciences study design

All studies must disclose on these points even when the disclosure is negative.

Sample size	Experiments repeated at least 2 independent times for two independently cultured individual cell lines
Data exclusions	N/A, all data included.
Replication	Multiple independent experiments with independently cultured individual cell lines
Randomization	N/A
Blinding	*Describe whether the investigators were blinded to group allocation during data collection and/or analysis. If blinding was not possible, describe why OR explain why blinding was not relevant to your study.*

Reporting for specific materials, systems and methods

We require information from authors about some types of materials, experimental systems and methods used in many studies. Here, indicate whether each material, system or method listed is relevant to your study. If you are not sure if a list item applies to your research, read the appropriate section before selecting a response.

Materials & experimental systems

n/a	Involved in the study	
☐	☒	Antibodies
☐	☒	Eukaryotic cell lines
☒	☐	Palaeontology and archaeology
☒	☐	Animals and other organisms
☐	☒	Human research participants
☐	☒	Clinical data
☒	☐	Dual use research of concern

Methods

n/a	Involved in the study	
☒	☐	ChIP-seq
☐	☒	Flow cytometry
☒	☐	MRI-based neuroimaging

Antibodies

Antibodies used	24 different primary antibodies used, full details provided in Supplementary Table 6 of manuscript
Validation	All primary antibodies, with the exception of the Stx5L-specific antibody, have been used in literature or validated by the manufacturer for the application (Suppl Table 6). The Stx5L-specific antibody was validated in this study on Stx5L-lacking HeLa cells generated with CRISPR/Cas9 (Supplementary Figure 13).

Eukaryotic cell lines

Policy information about cell lines

Cell line source(s)	HeLa cells from ATCC
Authentication	Short Tandem Repeat profiling by ATCC
Mycoplasma contamination	Cell lines were routinely tested and found negative for mycoplasma contamination
Commonly misidentified lines (See ICLAC register)	N/A

Human research participants

Policy information about studies involving human research participants

Population characteristics	Full details of Stx5M55V patients given in Supplementary Table 1.
Recruitment	Collected material from 3 patients with Stx5M55V mutations.
Ethics oversight	Research Ethics Committee of the University of Tartu: approval numbers 210/M-17, 212/M-31 and 235/M-13.

Note that full information on the approval of the study protocol must also be provided in the manuscript.

Clinical data

Policy information about clinical studies

All manuscripts should comply with the ICMJE guidelines for publication of clinical research and a completed CONSORT checklist must be included with all submissions.

Clinical trial registration	Study approved by Research Ethics Committee of the University of Tartu: approval numbers 210/M-17, 212/M-31 and 235/M-13
Study protocol	N/A
Data collection	Collected material from 3 patients with Stx5M55V mutations. Study approval dates 19.12.2011, 20.02.2012 and 17.03.2014
Outcomes	N/A

Flow Cytometry

Plots

Confirm that:

- [x] The axis labels state the marker and fluorochrome used (e.g. CD4-FITC).
- [x] The axis scales are clearly visible. Include numbers along axes only for bottom left plot of group (a 'group' is an analysis of identical markers).
- [x] All plots are contour plots with outliers or pseudocolor plots.
- [x] A numerical value for number of cells or percentage (with statistics) is provided.

Methodology

Sample preparation	HeLa cells on culture plates were released with 2 mM EDTA in PBS, fixed with 4% paraformaldehyde and stained using biotinylated lectins.
Instrument	Becton Dickinson Biosciences FACSLyric
Software	Data was acquired using BD FACSuite and analyzed with FlowJo.
Cell population abundance	Non-debris population was >90% of the parent, singlet population was >90% of the parent.
Gating strategy	Cells were gated on FSC-A/SSC-A to exclude debris, then gated on FSC-A/FSC-H to exclude doublets. An unstained control sample was used to discriminate positively stained cells from negative cells (see also Supplementary Figure 5).

- [x] Tick this box to confirm that a figure exemplifying the gating strategy is provided in the Supplementary Information.
